# Translational studies on pancreatic cancer and gastric cancer: a methodology in PhD thesis

**DOI:** 10.3389/fphar.2025.1604017

**Published:** 2025-05-30

**Authors:** Mathilde Resell, Gunnar Qvigstad, Timothy C. Wang, Anne S. Quante, África González-Fernández, Helge Waldum, Duan Chen, Chun-Mei Zhao

**Affiliations:** ^1^ Department of Clinical and Molecular Medicine, Norwegian University of Science and Technology, Trondheim, Norway; ^2^ Department of Medicine, St. Olavs University Hospital, Trondheim, Norway; ^3^ Department of Medicine and Irving Cancer Research Center, Columbia University Medical Center, New York, NY, United States; ^4^ Institute of Human Genetics, Technical University of Munich (TUM), TUM School of Medicine and Health, TUM University Hospital, Munich, Germany; ^5^ CINBIO, Universidade de Vigo, Vigo, Spain; ^6^ Instituto de Investigación Sanitaria Galicia Sur (IIS-GS), Hospital Alvaro Cunqueiro, Vigo (Pontevedra), Spain

**Keywords:** PhD research methodology, cancer, proteomics, knowledge discovery, patient and public involvement

## Abstract

Pancreatic ductal adenocarcinoma (PDAC) and gastric adenocarcinoma (GA) are aggressive cancers with poor prognoses, demanding innovative approaches to advance treatment strategies and prevention efforts. This article presents a methodology in connection with PhD thesis on PDAC and GA, including motivation and knowledge in literature (Paper I), various research models (Paper II), knowledge discovery (Papers III and IV), and thesis assessment and evaluation (dissertation). The four studies aimed to address the gaps between patients and researchers and between basic and clinical research. Patient and Public Involvement (PPI) was explored to align research priorities with patients’ needs. While PPI emphasized the importance of treatment-focused research, researchers and scientific journals prioritized basic science. Research guidance of “Findable, Accessible, Interoperable, and Reusable” (FAIR) was implanted in the studies, particularly proteomics datasets of different research models on PDAC. An analytic workflow for knowledge discovery with systems modeling was developed, leading to identification of translational targets of proteins and signaling networks on PDAC. Gastric intestinal metaplasia (GIM) is associated with GA. Multi-bioinformatics identified potential biomarkers for GA-related GIM, including genes and signaling networks. Potential repurposed drugs were also identified for both PDAC and GIM. In conclusion, the methodology was instructive in completing PhD thesis, whereas the findings in the original papers added new knowledge in translational research on PDAC and GA.

## 1 Introduction

Patient and Public Involvement (PPI) emphasizes the active involvement of patients and the public throughout the research process (Price et al., 2022; Aguayo et al., 2021; Roessler and Schmitt, 2021), fostering collaboration to improve research relevance and outcomes. Despite the value, there are potential gaps between patients and researchers to effectively enrich the research focus and objectives (Ludwig et al., 2020). Translational research aims to translate knowledge from fundamental sciences to practical applications in patient care (Seyhan, 2019; Drolet and Lorenzi, 2011; van der Laan and Boenink, 2015). However, the translational process is often labor-intensive and complex, requiring multiple stages of testing and refinement to ensure effective translation from bench to bedside (Schuhmacher et al., 2023). Systems modeling is a versatile tool applicable to a wide range of systems beyond biology (Lin and Chao, 2019). In cancer research, systems biology uses modeling to establish plausibility, characterize systems, and make clinical predictions.

**FIGURE 1:**
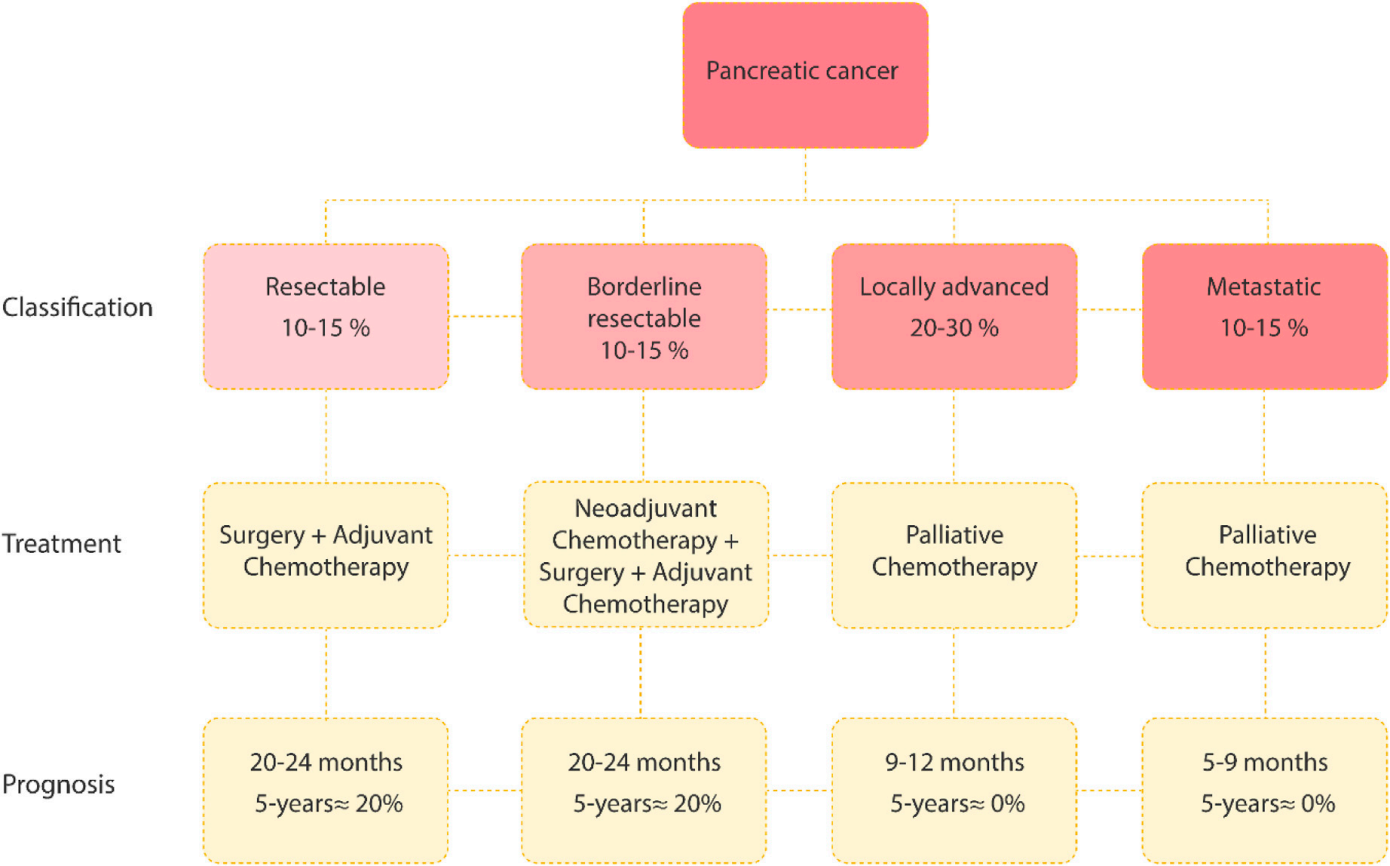
Classification, treatment strategies, and prognosis for pancreatic cancer. The disease can be categorized as resectable, borderline resectable, locally advanced, or metastatic, with corresponding survival rates. Resectable and borderline cases are treated with surgery and chemotherapy, offering a median survival of 20–24 months and a 5-year survival rate of ∼20%. Locally advanced and metastatic cases rely on palliative chemotherapy, with median survival of 9–12 months and 5–9 months, respectively, and no significant 5-year survival (Network, 2024). Created in Ilustrator.

Pancreatic cancer is a highly fatal malignancy, whereas pancreatic ductal adenocarcinoma (PDAC) accounts for more than 90% of all cases (Orth et al., 2019). The 5-year survival rate for PDAC remains poor, with about 13% (Ushio et al., 2021; Siegel et al., 2025). The treatment landscape for PDAC is challenging. Current therapies are primarily palliative, focusing on survival extension and symptom management rather than cure, especially in advanced stages (Kolbeinsson et al., 2023) (Figure 1).

The persistent high mortality rates, alongside only modest improvements in survival, underscore the urgent need for truly novel treatments for pancreatic cancer, as current strategies rely on the same chemotherapy drugs in varying combinations. This highlights a critical gap in therapeutic efficacy, as the limited options fail to significantly impact long-term survival or reduce mortality.

**FIGURE 2:**
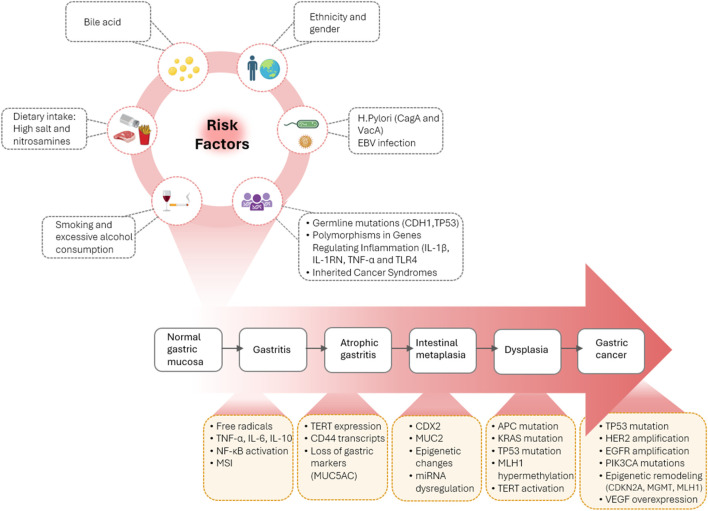
Key risk factors and molecular events in the progression of gastric cancer (GC). Risk factors include dietary intake, smoking, alcohol, bile acids, *H. pylori* (CagA, VacA), EBV, and genetic predispositions (e.g., CDH1, TP53 mutations, inflammation-related polymorphisms). The so-called Correa’s cascade progresses from normal mucosa to gastritis, atrophic gastritis, intestinal metaplasia, dysplasia, and GC. Each stage features distinct molecular changes. Created in Illustrator.

Gastric cancer (GC) ranks as the fifth most common cancer and is the fifth leading cause of cancer related deaths globally (Ferlay et al., 2024). Despite advances in treatments like immunotherapy and targeted therapies, the overall prognosis remains poor, particularly for those diagnosed at advanced stages (Guan et al., 2023; NCCN, 2024). Early detection and primary prevention strategies, such as *H. pylori* eradication, are crucial to controlling GC incidence and improving outcomes (NCCN, 2024; Hayakawa et al., 2021). Gastric Intestinal Metaplasia (GIM) is believed to be a precancerous condition, a process often driven by chronic inflammation by *H*. *pylori* infection, bile acid exposure, or environmental factors (Shah et al., 2020; He et al., 2022). This metaplastic shift has been described as the Correa’s cascade which proposes the progression from chronic gastritis to atrophic gastritis, then to GIM, and ultimately to gastric adenocarcinoma (GA) (Figure 2). However, it should be kept in mind that “association” in the Correa cascade should not be considered as “causality”. In other words, associations can arise between variables in the presence (i.e., GIM causes GA) and absence (i.e., they have a common cause) of a causal relationship.

American Gastroenterological Association (AGA), the European Society of Gastrointestinal Endoscopy and the British Society of Gastroenterology have recommended no specific treatments against GIM, except for the eradication of *H. pylori*. In general, it means “watch and wait,” namely, endoscopic surveillance with 3- and 5-year intervals. The latest guidelines entitled “The road to a world-unified approach to the management of patients with gastric intestinal metaplasia” suggested that there is an opportunity to enhance the research agenda in this field (Correction, 2024).

Drug repurposing provides a promising approach to expand treatment options for both PDAC and GIM. Systems biology strengthens this approach by providing an in-depth view of the molecular networks within the tumor and helping identify “hub” proteins. These proteins serve as central nodes that regulate multiple signaling pathways critical to cancer progression, making them high-value targets for therapeutic intervention. Targeting such hubs allows selective weakening of the oncogenic network; however, some hubs, due to their essential roles, cannot be safely targeted without affecting normal cells (Perrone et al., 2024; Rabben et al., 2021).

This “brief research report” presents not just a summary of four original research articles in the PhD thesis but a methodology of PhD education characterized as a “Method of Knowing” by including “Thinking” (motivation and knowledge in literature, Paper I); “Doing” (various research models, Paper II); and “Re-thinking” (knowledge discovery in datasets, Papers III and IV).

The scientific objective of this thesis was to address the translational gaps in PDAC and GA by identifying and validating biomarkers and therapeutic targets. Specifically, this work utilizes systems modeling with multi-bioinformatics to evaluate commonalities and differences across various PDAC and GIM research models, including cell lines, organoids, spheroids, and murine and human tissue samples. By integrating these models, this thesis aimed to uncover molecular targets that hold translational potential and are amenable to drug repurposing for both diseases. It should also be noticed that this work assesses the impact of patient and public involvement, aiming to align research priorities with patient needs to enhance the clinical relevance of findings. Through these methods, this thesis seeks to identify key biomarkers, pathways, and drug candidates to facilitate early detection and improve treatment efficacy, ultimately addressing the pressing needs in PDAC and GIM patient care.

Accordingly, the specific objectives are as follows:

Paper I: To identify gaps between the needs of end-users and the interests of researchers in pancreatic cancer research, particularly regarding research motivation and dissemination, and to evaluate the influence of PPI in the most-cited pancreatic cancer studies (Resell et al., 2024).

Paper II: To create and make public a comprehensive proteomic dataset across various PDAC models—including cell lines, spheroids, organoids, and murine and human tissues—to support the identification of biomarkers and therapeutic targets by the broader research community (Resell et al., 2025a).

Paper III: To develop a systems modeling framework to bridge translational gaps in PDAC research, focusing on identifying common proteins and pathways as potential targets for drug repurposing [Revised version (Resell et al., 2025b)].

Paper IV: To use multi-bioinformatics analysis to identify biomarkers and potential drug repurposing targets that could prevent the progression of gastric intestinal metaplasia to gastric adenocarcinoma (Andersen et al., 2024).

## 2 Methods

**FIGURE 3:**
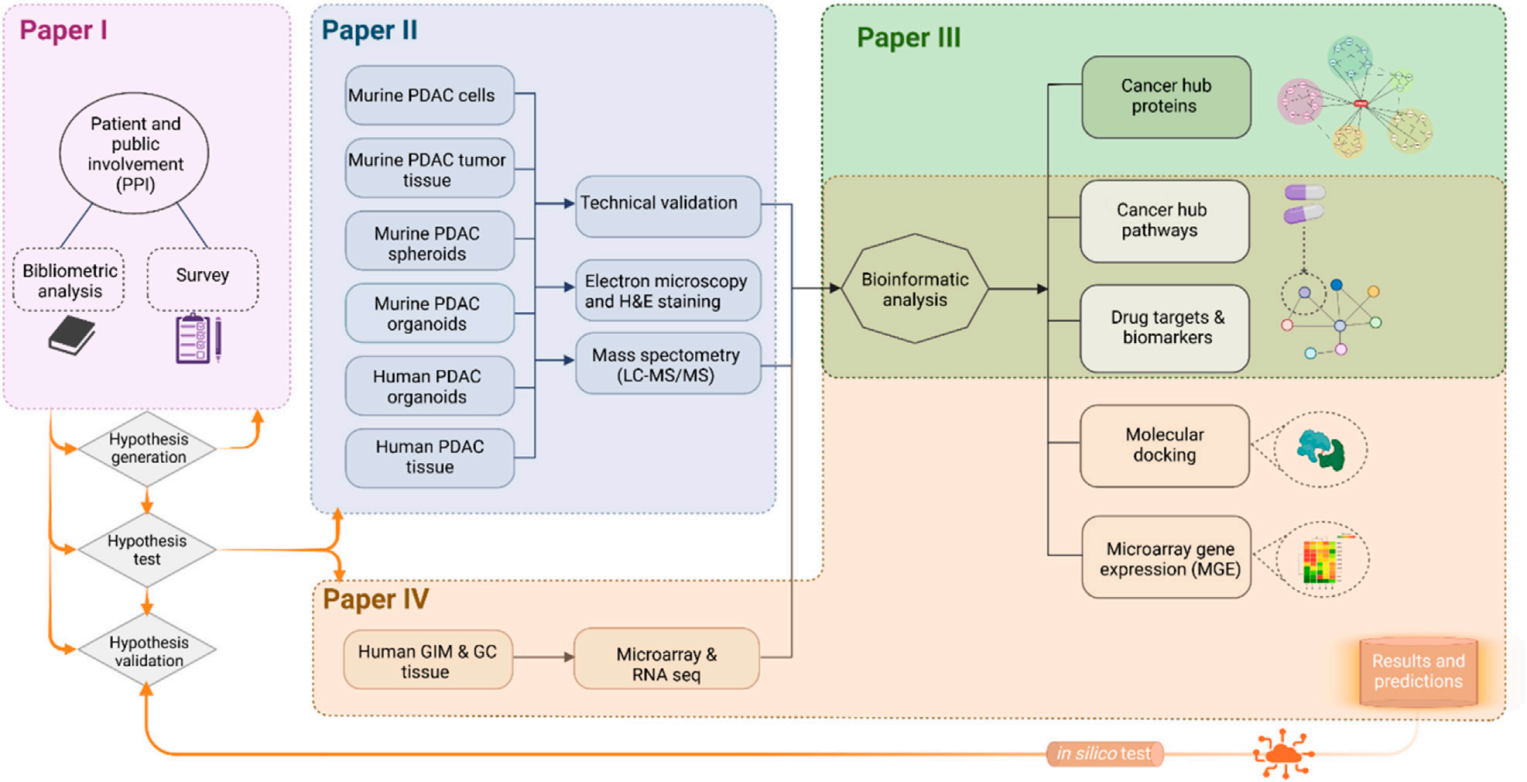
Thesis design. This thesis integrates patient and public involvement (PPI) to align research priorities with end-user perspectives. Paper I explores end-user interests in pancreatic cancer research, compared to researchers’ priorities. Paper II presents proteomic data from six preclinical models of pancreatic cancer, validated for relevance and made publicly accessible to guide model selection. Paper III applies systems biology to compare these models with patient data, identifying hub proteins and key pathways. Paper IV focuses on transcriptomic data from GIM and GA patients to identify biomarkers and explore drug repurposing opportunities for gastric cancer prevention. Created in BioRender.

The original aim of the PhD thesis was to initiate research from an end-user perspective to identify their interest in research focusing on pancreatic cancer, examined in Paper I. This approach included patients, close family and others and their interests were compared with researchers’ interests. The initiation of preclinical research requires selection of a research model that closely mimics the *in vivo* conditions, in this case PDAC patients. A hypothesis was then formulated to identify which research models for studying pancreatic cancer and gastric cancer have the highest relevance to patients. In Paper II, proteomic data from six different research models were presented, along with validation of each model. These datasets were made publicly accessible to aid others in selecting the most appropriate models for their specific aims. In Paper III, an analytic approach was applied to compare the proteomic profiles of all six research models. Using systems biology, the study integrated omics data and bioinformatic tools to identify similarities and disparities with patient data, uncovering central hub proteins and key canonical pathways. A similar systems biology approach was used in Paper IV, which utilized comprehensive omics analyses to identify potential biomarkers for GA-related GIM and predict drug repurposing opportunities for GA prevention. In this case, bioinformatic tools were applied exclusively to transcriptomic data from patient samples. The systems biology workflow and preliminary results of the PDAC preclinical research were presented at events such as a gathering organized by the Norwegian Pancreas Network with a focus on maintaining PPI integrity throughout the research process (Figure 3).

Transcriptomics and proteomics were used, and data analysis was performed by using Ingenuity Pathway Analysis (IPA), Cytoscape and molecular docking.

The research involving human subjects was approved by the Regional Committees for Medical and Health Research Ethics in central Norway (REK 2012-1029, REK OSLO Paper II), following the principles of Good Clinical Practice (GCP). Most experiments were conducted using spheroids, organoids, and cultured cells, minimizing the reliance on animal models. When animal studies were essential, they were carried out following the 3R principles—Reduce, Reuse, and Refine—to ensure humane and ethical treatment. These animal experiments were also approved by the Norwegian Medicines Agency and supervised by The Norwegian Food Safety Authority (Mattilsynet) (FOTS 7961).

The Research Council of Norway has established the policy for open science which includes (i) participation, involvement and citizen science (as did in Paper I; (ii) reforming research assessment; (iii) making research data FAIR (Findable, Accessible, Interoperable and Reusable) (as Paper II); (iv) Data infrastructure and European Open Science Cloud; and (v) Open access to publications and Plan S (as Papers I-IV).

## 3 Results

### 3.1 Gaps between PPI and researchers

**FIGURE 4:**
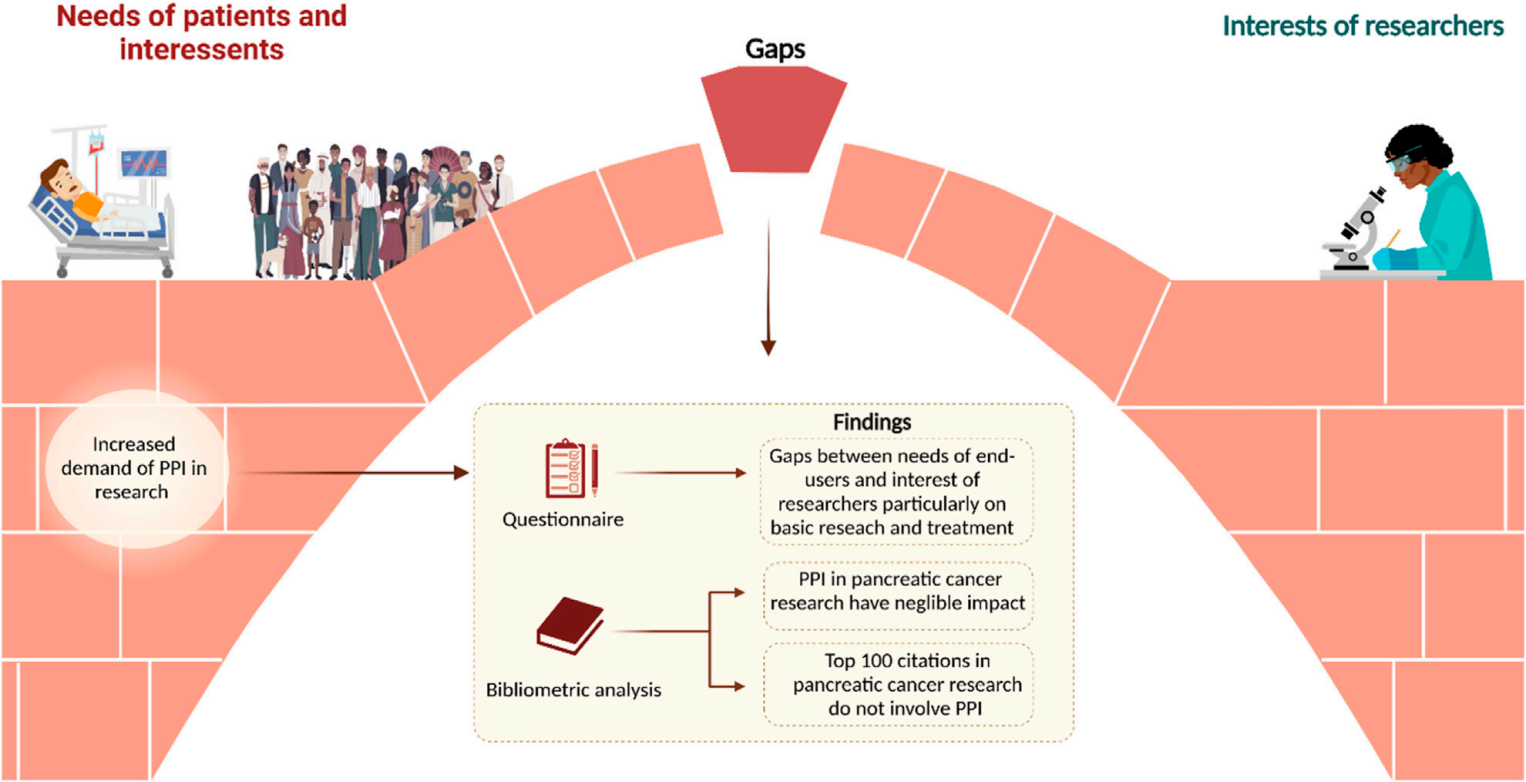
Graphic summary of PPI results of Paper I. Of note, gaps were identified from questionnaire and literature analysis.

In Paper I, the questionnaire showed gaps in how end-users and researchers value different aspects of research. Specifically, patients attributed greater importance to treatment compared to researchers, while researchers assigned more weight to basic research. Moreover, the analysis of the top-cited literature showed that PPI was almost entirely absent, contributing only 0.1% of all studies on pancreatic cancer, suggesting a systemic undervaluation of patient perspectives in driving research priorities (Figure 4).

### 3.2 FAIR for PDAC

**FIGURE 5:**
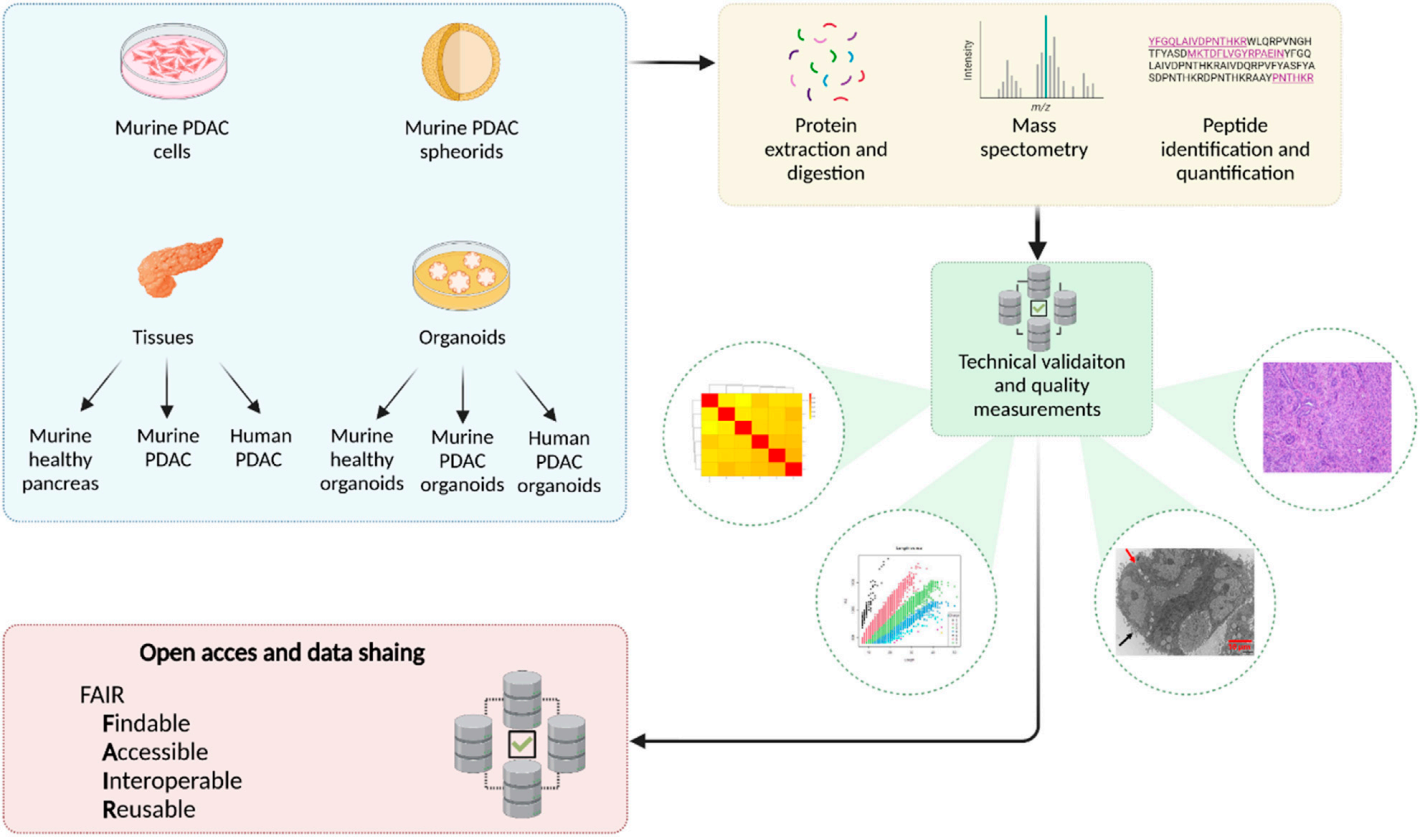
Graphic summary of FAIR results of Paper II. Of note, eight datasets of proteomics are in FAIR.

As the purpose of Paper II was to make contributions to the database of proteomics, the datasets of mass spectrometry-based proteomics of various models, including PDAC cell lines, spheroids, organoids, and tissue samples derived from both murine and human sources were made according to FAIR (Figure 5). Detailed preparation and technical quality control were presented, particularly focusing on bridging the translational gap between preclinical PDAC research models and clinical applications, thereby advancing collective ability to improve early detection and develop effective treatment strategies for PDAC.

### 3.3 Systems modeling and translational targets for PDAC

**FIGURE 6:**
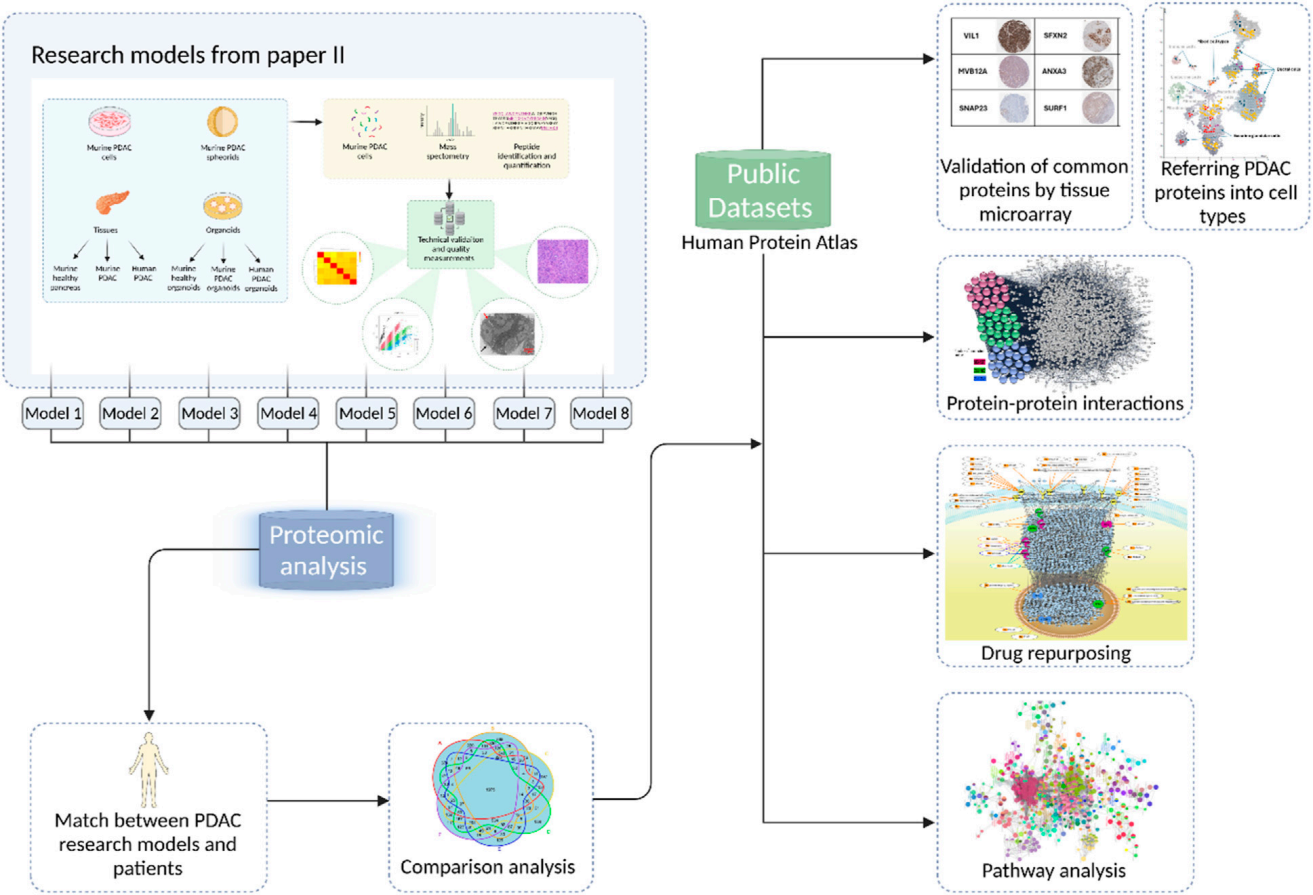
Graphic summary of systems modeling of Paper III. Of note, matched proteins between PDAC research models and patients were identified for further biomarkers and drug targets (including drug repurposing).

In Paper III, a “systems modeling” workflow was introduced for knowledge discovery using the datasets presented in Paper II (Figure 6). This framework integrated proteomics and bioinformatics to examine protein profile across diverse models. Of note, the samples sizes were limited in some models, which would reduce the statistical power when performing analysis of the differences between the models. The proteomics studies were designed to detect which proteins were presented rather than abundance/expression levels in attempt to show the match targets between research models and patients. The study identified 1,975 common proteins, revealing a 50%–60% overlap with PDAC tissues (Figure 6). Key hub proteins, such as GAPDH and HSP90AA1, were implicated in critical signaling pathways relevant to PDAC progression. Furthermore, this integrated workflow facilitated the identification of translational targets, addressing the long-standing translational gap in PDAC research. The findings support future investigations into personalized therapeutic strategies targeting these signaling networks.

### 3.4 Biomarkers for GA-related GIM

**FIGURE 7:**
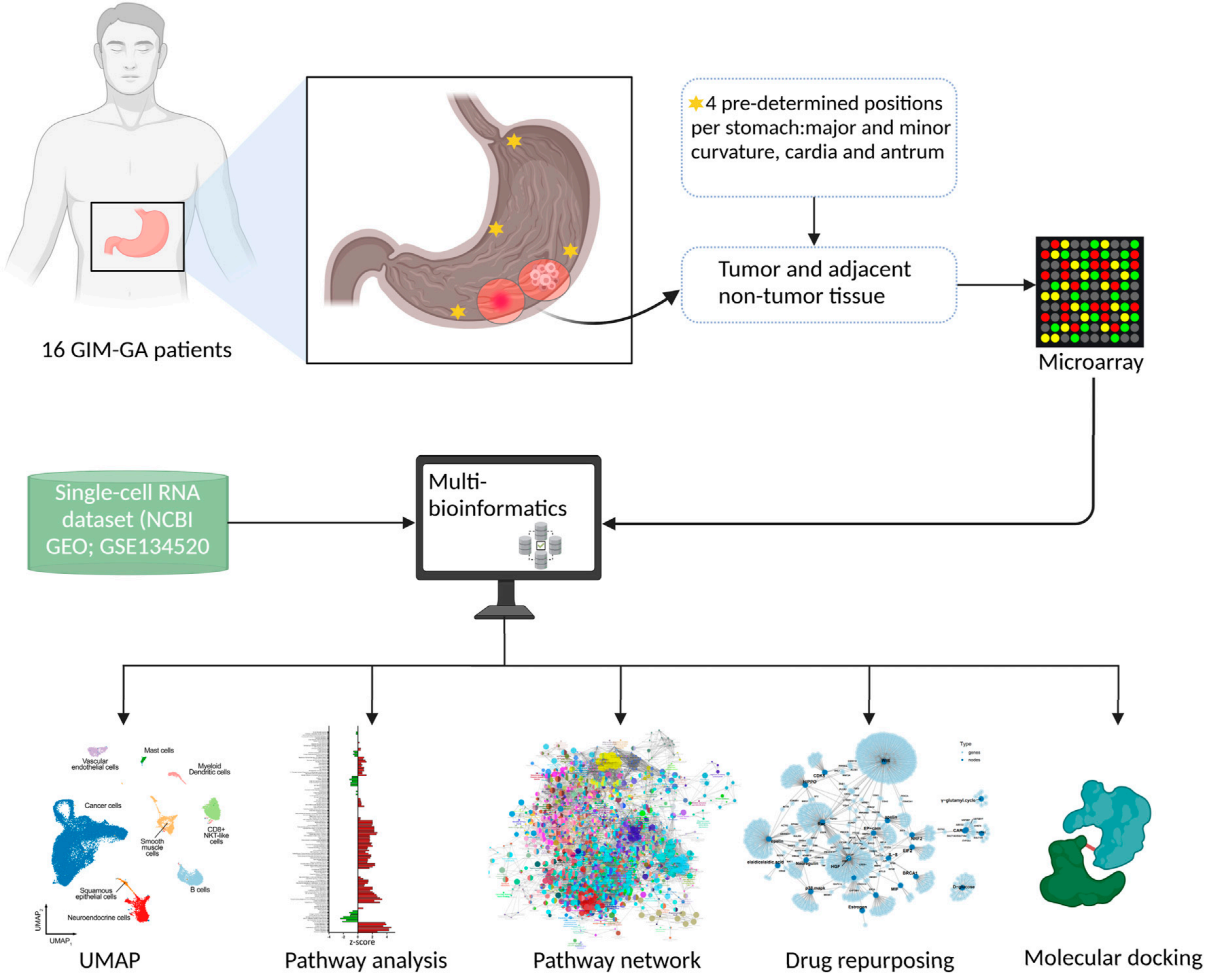
Graphic summary of multi-omics for discovery of biomarkers of Paper IV. Of note, multi-bioinformatics was applied to identify potential biomarkers and drug development.

In Paper IV, multi-bioinformatics approaches were used to identify potential biomarkers associated with the progression from GIM to GA, and to predict potential drugs that could be repurposed for GA prevention (Figure 7). Potential key biomarkers included genes such as RBP2 and CD44 that were differentially expressed between GIM and GA, and critical signaling pathways like Wnt and IL-22 signaling that may contribute to the transformation process. Potential drug targets, including epidermal growth factor receptor (EGFR) and tumor suppressor protein p53, which could serve as therapeutic candidates to mitigate malignant progression. Furthermore, molecular network and network of signaling pathways were constructed to be implicated in the progression of GIM to GA, providing an extensive set of potential targets for future therapeutic strategies.

## 4 Discussion

### 4.1 New methods to bridge the gaps in translational research

The gap in perspectives between patients and researchers regarding research priorities might negatively impact the translation of basic research findings into clinical research in terms of the value chain of “return on investment” by the two sides. PPI prioritizes how to get early detection and better quality of life, and avoid risk factors, whereas (basic) researchers often focus on the quality of their articles that can be recognized in scientific societies and funding bodies. To overcome the gaps, both researchers and scientific societies and journals should adherent to the frameworks like GRIPP2 (Guidance for Reporting Involvement of Patients and Public), requiring authors to disclose PPI activities, and scientific conferences could include sessions dedicated to patient engagement. These initiatives would signal the importance of PPI to the research community and encourage broader adoption. We believe that the impact of PPI can be enhanced by prestigious conferences and journals in consideration of publishing policies and encouragements. Of note, the study (Paper I) was presented to end-users (patients and their relatives) at the seminar arranged by the Pancreatic Cancer Network Norway and the Norwegian Cancer Society on World Pancreatic Cancer Day, the 16th of November 2023, but rejected by Digestive Disease Week (DDW 2023) and United European Gastroenterology (UEG Week 2023) [The other studies (Papers II–IV) were selected for presentations at DDW and UEG].

Furthermore, the researchers should follow the FAIR Principles for scientific data management and stewardship to accelerate the pace of scientific discovery. It should also be kept in mind that the gaps between the patients and researchers are understandable. Scientists are correct to prioritize basic science, as the reason better therapies have not emerged is that we still do not understand these cancers well enough, which should be highlighted in PPI.

The “systems modelling” can be defined as the process of developing abstract models of a system, with each model presenting a different view or perspective of that system, particularly in connection with “systems biology.” The “systems modeling” consists of study hypothesis, composition model (different experimental models), data processing model (i.e., proteomics), data-analysis model (interactions of omics), classification model (showing how entities have common characteristics), knowledge discovery model (data mining on hub proteins, protein-protein interactions and signaling pathways), results/prediction model, and feedback loops/interactions in-between. Of note, the systems modeling was enriched by including “objective filter,” “matched filter,” “knowledge filter,” and “hypothesis filters,” as the hypothesis was the “mismatch” with PDAC patients in translational research and the cancer cells as the targets rather than normal pancreas.

In the systems modeling workflow, public datasets, such as the single-cell atlas of GIM/GA and the human protein atlas, cand be integrated. A “knowledge filter” which acts as a “human-in-the-loop” element is employed for interpreting data, leading to the generation of new hypotheses. Notably, data mining and knowledge discovery in databases can be applied to cluster data and filter out or eliminate “noise.” The systems modeling approach can be highly adaptable, enabling the integration of additional models and filters tailored to specific hypotheses. These may include human cell lines, genetically engineered mouse models, components of the tumor microenvironment such as fibroblasts and cancer-associated fibroblast organoids, and blood samples.

### 4.2 New knowledge to overcome the gaps between research models in translational research

The mismatch between research models and human patients is a multifaceted issue involving genetic, physiological, and technical differences. For instance, the average rate of successful translation from animal models to clinical cancer trials is less than 8% and about 90% of clinical drug development fails (Mak et al., 2014; Sun et al., 2022). Addressing these discrepancies requires the development of more accurate and reliable models, improved cell line authentication, and the use of humanized models to bridge the gap between pre-clinical research and clinical application (De Jong and Maina, 2010; Shipley et al., 2016; Tomasin et al., 2019; Ye et al., 2015). In Papers II and III, identifying matched proteins across different research models, such as the five PDAC models compared with human tissues, was a crucial step in bridging the translational gap between laboratory research and clinical applications. The significance of these approaches lies in its ability to enhance the relevance of preclinical findings. By identifying proteins consistently expressed in human tissues and models, the study reduces the risk of focusing on artifacts that do not translate to patient outcomes. This provides a strong foundation for identifying therapeutic targets, biomarkers, and pathways that are more likely to hold clinical significance. Of note, the importance of not relying solely on assumptions about model fidelity based on their origin (mouse vs. human) but instead leveraging comparative proteomics to identify the most suitable model for specific research objectives.

Hub proteins are central to the architecture and functionality of protein-protein interaction (PPI), acting as critical regulators of cellular processes and potential therapeutic targets in diseases like PDAC and GA-related GIM (Zhang et al., 2018). In both cases as shown in Papers III and IV, β-catenin in WNT signaling pathway and CD44 were identified as a top hub protein and could be potential biomarkers and drug targets. As a next step, exploring *in silico* drug modeling could help prioritize the identified therapeutic targets and design selective inhibitors tailored to PDAC or GA-related GIM. This approach would enable the simulation of drug-target interactions, offering insights into more specificity and potential efficacy while minimizing off-target effects. Such computational efforts, combined with experimental validation, could accelerate the development of targeted therapies, particularly for complex targets like β-catenin and CD44. CD44 appears particularly significant as it holds particular importance due to its role in the WNT/β-catenin signaling pathway, which is frequently implicated in cancer stem cell regulation and tumor initiation. CD44 stabilizes β-catenin, allowing it to accumulate in the nucleus and drive the expression of stemness-related genes and proliferative targets (Hayakawa et al., 2021; Takaishi et al., 2009).

### 4.3 Future perspectives

The integration of advanced technologies holds significant potential for advancing systems modeling in cancer research. Artificial Intelligence (AI) and Machine Learning (ML) can efficiently analyze large multi-omic datasets, identifying the networks of signaling pathways as predictive biomarkers and therapeutic targets. Knowledge discovery in databases (KDD) can integrate multi-omics and clinical datasets to reveal critical pathways and shared molecular features. The concept of digital twins, creating virtual patient-specific models, could revolutionize personalized medicine by simulating disease progression and predicting therapeutic responses. Leveraging these tools can bridge the translational gap, enabling early detection, targeted therapy, and improved outcomes in PDAC and GA-related GIM.

### 4.4 Evaluation by the assessment committee

The Faculty of Medine and Health Sciences of Norwegian University of Science and Technology requests the committee to consider whether the thesis is an independent and comprehensive piece of work of high academic standards; to consider the methodical, theoretical and empirical bases, documentation, treatment of literature and form of presentation in the thesis; to consider whether the materials and methods applied are relevant to the issues raised in the thesis, and whether the arguments and conclusions posited are tenable, and to determine if the thesis contributes to new knowledge to the discipline. It requests to give the evaluation of each paper that were included in the thesis, and to consider to what extent the candidate’s contribution to joint publication can be identified and whether the candidate is solely responsible for a sufficient part of the thesis.

The assessment committee has evaluated the thesis accordingly. In general, the thesis used modern techniques, different biocomputing programs, especially in transcriptomic analysis. The materials and methods were relevant to the issues raised in the thesis. The arguments and conclusions were supported by the results. However, it could be better to present the literature particularly in comparison with the results of this thesis. Paper I was interesting but not clear how they will implement the recommendations in the future work. In Paper II, the effort of making proteomics to different samples tried to find common elements in all those models, but large differences were found between them, making it difficult to know which could be the best for studying a scenario more like human pancreatic cancer. The weakness was that few samples of each model were analyzed, decreasing the statistical power. Paper III was interesting and well performed although with some limitations as the authors stated. Paper IV was very interesting work, identifying potential pathways in pre-tumoral lesions, showing potential biomarkers and therapeutic targets. In conclusion, the thesis was worthy of defense without changes.

During the dissertation, additional comments and suggestions were raised, such as further analysis of mismatched proteins among the research models, plasma biomarkers for screening, early diagnosis, and qualitative vs*.* quantitative analysis. Regarding the organoid models, molecular validation of PDAC and growth process from initial cell aggregation, proliferation, migration to differentiation should be concerned. The Correa cascade is a hypothesis that still needs to be validated.

## Data Availability

The raw data supporting the conclusions of this article will be made available by the authors, without undue reservation.
